# A novel MCF-10A line allowing conditional oncogene expression in 3D culture

**DOI:** 10.1186/1478-811X-9-17

**Published:** 2011-07-13

**Authors:** Ricarda Herr, Franziska U Wöhrle, Christina Danke, Christian Berens, Tilman Brummer

**Affiliations:** 1 Centre for Biological Systems Analysis (ZBSA), Albert-Ludwigs-University Freiburg, Habsburgerstraße 49, 79104 Freiburg, Germany; 2 Institute for Biology III, Faculty of Biology, Albert-Ludwigs-University Freiburg, Schänzlestraße 1, 79104 Freiburg, Germany; 3 Spemann Graduate School of Biology and Medicine, Albertstraße 19A, 79104 Freiburg, Germany; 4 Centre for Biological Signaling Studies BIOSS, Hebelstraße 25, 79104 Freiburg, Germany; 5 Comprehensive Cancer Center Freiburg (CCCF), Universitätsklinikum Freiburg, Hugstetter Straße 55, 79106 Freiburg, Germany; 6 Division of Microbiology, Department of Biology, Friedrich-Alexander-University Erlangen-Nuremberg, Staudtstraße 5, 91058 Erlangen, Germany

**Keywords:** MCF-10A, mammary epithelium, carcinogenesis, *BRAF*, epithelial-mesenchymal transition (EMT), tetracycline-inducible gene expression system, apoptosis, E-Cadherin, KI-67, Caspase-3

## Abstract

**Introduction**

Non-transformed mammary epithelial cell lines such as MCF-10A recapitulate epithelial morphogenesis in three-dimensional (3D) tissue culture by forming acinar structures. They represent an important tool to characterize the biological properties of oncogenes and to model early carcinogenic events. So far, however, these approaches were restricted to cells with constitutive oncogene expression prior to the set-up of 3D cultures. Although very informative, this experimental setting has precluded the analysis of effects caused by sudden oncoprotein expression or withdrawal in established epithelial cultures. Here, we report the establishment and use of a stable MCF-10A cell line (MCF-10Atet) fitted with a novel and improved doxycycline (dox)-regulated expression system allowing the conditional expression of any transgene.

**Methods**

MCF-10Atet cells were generated by stable transfection with pWHE644, a vector expressing a second generation tetracycline-regulated transactivator and a novel transcriptional silencer. In order to test the properties of this new repressor/activator switch, MCF-10Atet cells were transfected with a second plasmid, pTET-HA*BRAF*-IRES-GFP, which responds to dox treatment with the production of a bi-cistronic transcript encoding hemagglutinin-tagged B-Raf and green fluorescent protein (GFP). This improved conditional expression system was then characterized in detail in terms of its response to various dox concentrations and exposure times. The plasticity of the phenotype provoked by oncogenic B-Raf^V600E ^in MCF-10Atet cells was analyzed in 3D cultures by dox exposure and subsequent wash-out.

**Results**

MCF-10Atet cells represent a tightly controlled, conditional gene expression system. Using B-Raf^V600E ^as a model oncoprotein, we show that its sudden expression in established 3D cultures results in the loss of acinar organization, the induction of an invasive phenotype and hallmarks of epithelial-to-mesenchymal transition (EMT). Importantly, we show for the first time that this severe transformed phenotype can be reversed by dox wash-out and concomitant termination of oncogene expression.

**Conclusions**

Taken together, we have generated a stable MCF-10A subline allowing tight dox-controlled and reversible expression of any transgene without the need to modify its product by introducing artificial dimerization or ligand-binding domains. This system will be very valuable to address phenomena such as EMT, oncogene addiction, oncogene-induced senescence and drug resistance.

## Background

The majority of human cancers (carcinomas), including breast cancer, are caused by the malignant transformation of epithelial cells [1]. Epithelia form well-ordered sheets with well-defined planar and apico-basal polarity axes [2,3]. As they are surrounded by a basal lamina, they constitute a critical barrier between the internal milieu of the body and the exterior space. They also separate the secretory from the stromal compartment in glandular tissues such as the breast, prostate or pancreas. The proper orchestration of proliferation and differentiation processes as well as the development of the aforementioned polarity axes is key to the biological functions of epithelia. Conversely, the progressive loss of this well-ordered architecture is a hallmark of tumors of epithelial origin and has been used by pathologists for carcinoma classification since decades. Until recently, cell biologists have studied the architecture and differentiation of epithelia either *in vivo *or have resorted to *in vitro *model systems in which epithelial cells were grown as a monolayer on artificial surfaces such as plastic culture dishes. Notably, not more than a decade ago, several laboratories studying epithelial cells and their transformed counterparts began to grow epithelial cells in three-dimensional (3D) culture systems, which recapitulate many facets of epithelial tissues *in vivo *[1,4-6]. For example, the immortalized, non-transformed cell line MCF-10A retains the intrinsic ability of mammary epithelial cells (MECs) to undergo acinar morphogenesis in 3D matrigel cultures, a process that relies on growth-factor-dependent proliferation, the induction of luminal programmed cell death, establishment of an apico-basal polarity axis and the deposition of a basal lamina [1,3,7,8]. Studies from various laboratories have shown that various oncogenes can have distinct perturbing effects on acinar morphogenesis and induce supra-cellular effects in the cell colonies, which are also observed by pathologists in neoplastic lesions such as ductal carcinoma *in situ *(DCIS) [1]. For example, purely "proliferative" oncogenes enhance acinar size, while mainly "anti-apoptotic" oncogenes block or delay luminal clearance [7,9]. Very potent oncogenes such as activated forms of the receptor tyrosine kinase (RTK) HER2/ErbB2 impinge on multiple aspects of acinus development by enhancing not only the proliferation of oncogene-expressing cells, but also by blocking luminal cell death and by disturbing mechanisms establishing cellular polarity [7,10]. So far, however, these studies have used single cells already expressing the oncogene-of-interest prior to their seeding into matrigel. In stark contrast, however, carcinomas arise from individual, somatically mutated cells within the context of an established epithelium. Consequently, it would be important to have a system available in which oncogene expression can be switched on and off at will during acinar morphogenesis. To the best of our knowledge such a system does not exist for human mammary epithelial cells, as has also been noted by others [11]. The only exceptions are reports by the Muthuswamy and Brugge laboratories, which have used genetically modified ErbB receptors, which can be activated by coumermycin-induced dimerization. Although extremely informative, this system has limitations as it requires the oncoprotein of interest to tolerate the fusion partner and it relies on the fact that the activation of the protein in question occurs upon dimerization. Similarly, fusion proteins between a modified ligand-binding domain of the estrogen receptor (ER) and the protein of interest have been used in MECs to control the activity or subcellular localization of a protein of interest as it has been reported for the catalytic domains of Akt or Raf-proteins [9,12,13]. However, these approaches, despite the fact that they respond rapidly to the inducer 4-hydroxytamoxifen [4-HT], are often hampered by a certain degree of leakiness, e.g. due to partial degradation of the fusion protein, and are also potentially limited by the presence of the ER domain. Here, we report the generation and application of a versatile MCF-10A subline, which allows the tightly regulated and efficient expression of any oncogene of interest. Using B-Raf and its oncogenic form (B-Raf^V600E^) as a model oncoprotein, we also provide an example as to how these cells and their transgene expression level can be efficiently monitored and tracked in real time.

## Results

### Generation of a stable MCF-10A cell line containing an improved Tet-regulated expression system

**Figure 1:**
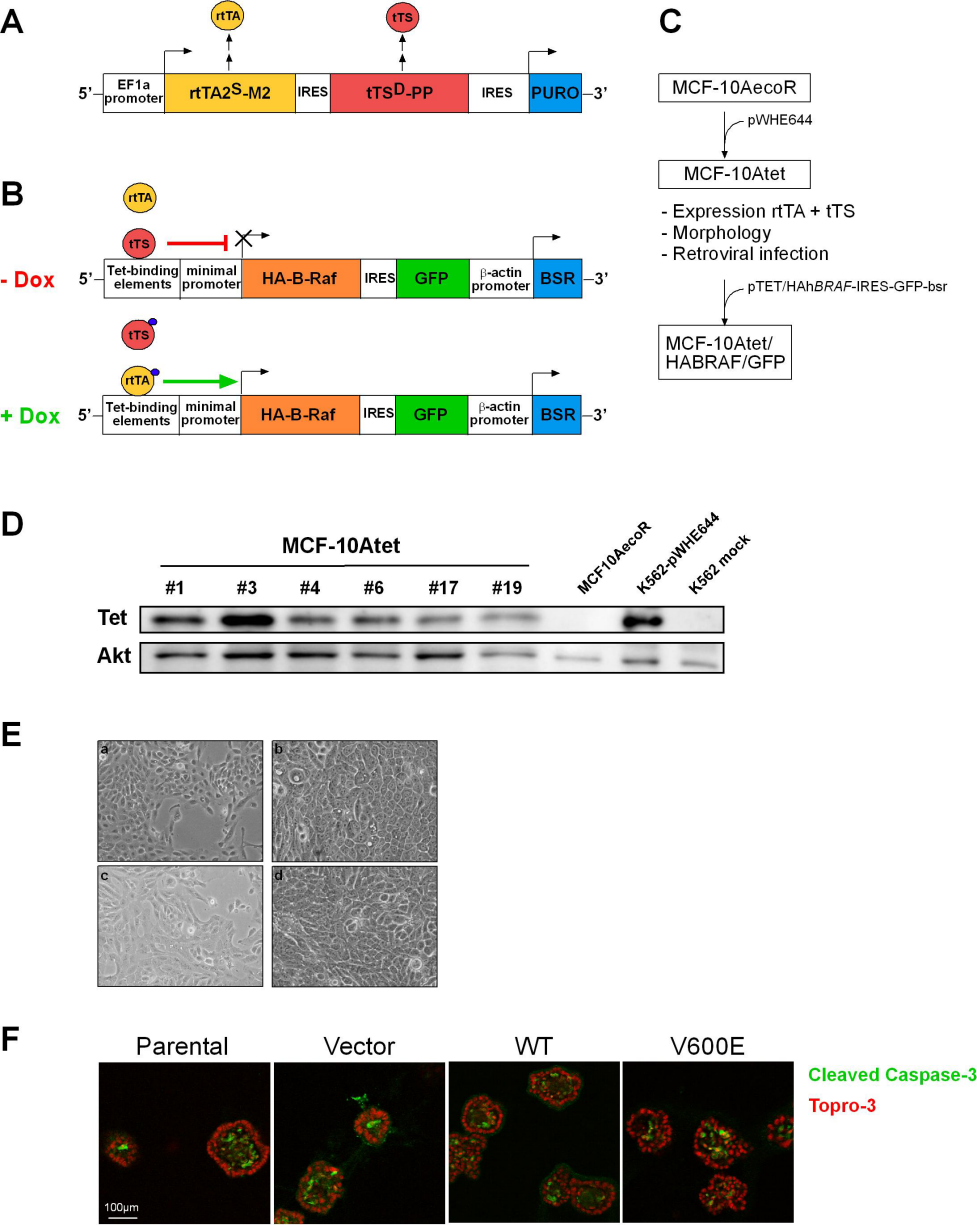
**Establishment of a Tet-regulated expression system for MCF-10A cells**. **(**A**)** Schematic representation of pWHE644 and** (B) **pTET/HAh*BRAF*-IRES-GFP-bsr. In the absence of dox, the activator protein (yellow) displays no DNA binding activity and the repressor protein (red) binds to the expression vector, suppressing expression of the gene-of-interest. Upon dox treatment, the repressor looses, while the activator gains DNA binding activity and drives the transcription of the bi-cistronic HA-B-Raf-IRES-GFP mRNA. See *Methods *for further details. **(**C**)** Flow chart for the generation of Tet-inducible MCF-10A cell lines. **(**D**) **Western blot analysis for screening purposes showing the expression of both transregulator proteins in a series of independent MCF-10AecoR pools transfected with pWHE644 (MCF-10Atet). A lysate from K562 cells (Wöhrle et al, in preparation) expressing the rtTA and tTS proteins serves as positive control (K562-pWHE644). Parental MCF-10AecoR cells and mock transfected K562 cells serve as negative controls. Detection of AKT serves as loading control. Pool 19 was chosen for further evaluation and ultimately for transfection with the pTET-bsr constructs **(**E**)** Morphological comparison of MCF-10AecoR cells (a/b) and the cell pool 19 expressing the rtTA/tTS proteins (c/d) in the subconfluent state and grown to confluency, respectively. **(**F**)** Phenotypic comparison of parental MCF-10A cells [55] in 3D culture with three independent sublines of MCF-10Atet cells (sublines will be explained in detail below) at day 15. Note that all four sublines form acini displaying ongoing luminal clearance as indicated by the high number of cells stained with an antibody for cleaved caspase-3.

We decided to apply a novel version of a tetracycline (Tet) -inducible expression system to MCF-10A cells. Therefore, we transfected the MCF-10AecoR subline [14] with pWHE644 [15], a vector expressing a tri-cistronic transcript encoding (i) a second generation Tet-regulated transcriptional transactivator (rtTA2^S^-M2) lacking background activity and with increased sensitivity towards its effectors, like the tetracycline derivative doxycycline (dox) [16], (ii) a transcriptional silencer (tTS^D^-PP) with improved reactivation of gene expression in the presence of effectors (Danke *et al*., manuscript in revision) and (iii) puromycin acetylase as selection marker (Figure 1A). This expression cassette ensures that the rtTA2^S^-M2 and tTS^D^-PP proteins are efficiently co-expressed in puromycin-resistant cells, thereby providing tight regulation of artificial promoters with *tetO *elements responsible for Tet-binding (Figure 1B) [17]. This combination of Tet-responsive proteins ensures tight repression of transgene expression in the absence of the inducer without compromising the responsiveness of the *tetO *element containing construct [15]. A direct comparison of the novel regulatory system with the previously used containing a KRAB-based trans-silencer [18] revealed even tighter regulation in Jurkat cells, which contained stably integrated transregulators and which were transiently transfected with a Tet-responsive reporter plasmid (Additional data file 1). Furthermore, we have chosen the MCF-10AecoR subline as it allows the efficient infection with ecotropic retroviruses [14], which is a useful aspect for the subsequent introduction of additional transgenes. MCF-10AecoR cells stably transfected with pWHE644, termed MCF-10Atet in the following (Figure 1C), were then analyzed for transgene expression by Western blotting (Figure 1D). MCF-10Atet cells with prominent rtTA2^S^-M2 and tTS^D^-PP expression were further characterized to ensure that properties, like their susceptibility to ecotropic retroviruses (data not shown), the maintenance of their epithelial character (Figure 1E) and their ability to generate proper acini in 3D culture were not affected (Figure 1F). The latter aspect was investigated using a staining for cleaved caspase-3, which reports luminal apoptosis, a key event in acinar development integrating various morphogenetic processes [1,7].

**Figure 2:**
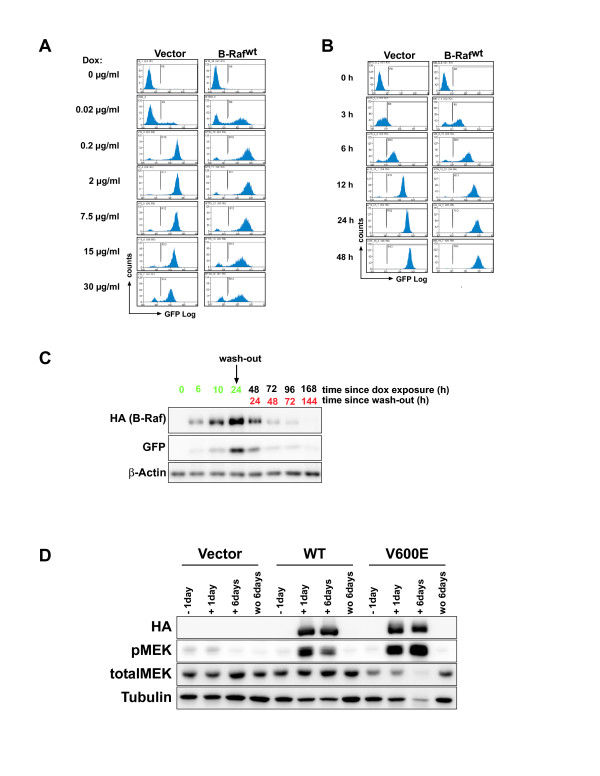
**Characterization of Tet-regulated transgene expression in MCF-10A cells**. **(A, B) **FACS analysis showing a typical **(A) **dose-response and **(B) **time course experiment. **(C) **MCF-10Atet cells stably transfected with pTET/HAh*BRAF*-GFP-bsr were induced with dox for 24 h followed by a wash-out of dox. Transgene expression was monitored by Western blotting.** (**D**)** MCF-10Atet cells were induced with dox for 24 h and either kept under dox treatment for additional 6 days (+ 6 days) or subjected to a dox wash-out (wo 6 days). Cells were lysed and Western blot analysis was performed using the indicated antibodies.

The Ser/Thr-kinase B-Raf represents an important signaling element of the Ras/Raf/MEK/ERK pathway [19,20]. This signaling cascade is often dysregulated in human cancer and was recently identified as the common denominator of signaling pathways associated with breast cancer risk in a genome wide association study [21]. Furthermore, although mutations in the *RAS *or *BRAF *genes are rarely observed in the overall majority of breast cancers, they occur more frequently in cell lines derived from basal type breast cancers, a rare, but difficult to treat subtype [22,23]. The importance of this pathway for this tumor entity is also illustrated by recent findings showing that basal type breast cancer cell lines are particularly susceptible towards growth inhibition by MEK inhibitors [24]. As we have previously shown that B-Raf signaling influences various aspects of MCF-10A biology [25], we decided to explore the role of B-Raf in these cells in more detail and tested the suitability of our inducible expression system using this proto-oncogene product. To this end, a representative population of the MCF-10Atet cells fulfilling the aforementioned criteria was transfected with either the plasmid pTET/HAh*BRAF*-IRES-GFP-bsr or pTET/HAh*BRAF*^V600E^-IRES-GFP-bsr (Figure 1C). The latter encodes for the most common B-Raf oncoprotein found in human tumors [26], while the former encodes for wildtype B-Raf (B-Raf^wt^). Given the potency of *BRAF*^V600E ^to transform MCF-10A cells in 2D and 3D culture [25], this oncogene is ideally suited to test the tightness and reversibility of our conditional system in 2D and 3D culture. Both vectors respond to dox administration with the expression of a bi-cistronic transcript encoding hemagglutinin-tagged human B-Raf (HAhB-Raf) and green fluorescent protein (GFP) (Figure 1B). The GFP fluorescence allows for the identification of dox-responsive cells and for sorting cell populations with desired transgene expression levels as described previously [14]. In addition, we transfected cells with pTET/GFP-bsr as an empty vector control. Stably transfected clones were then characterized in terms of leakiness and responsiveness to dox. First, we determined the optimal dox concentration by performing dose-response experiments. To this end, we used MCF-10Atet cells transfected with either the "empty" pTET/IRES-GFP or the pTET/HAh*BRAF*-IRES-GFP-bsr constructs, as we have shown previously that the expression of B-Raf^wt ^and GFP is well-tolerated in MCF-10A cells and does not induce dramatic phenotypic alterations as B-Raf^V600E ^[25]. As shown in Figure 2A, the expression of the HAh*BRAF*-IRES-GFP cassette is efficiently repressed in the absence of dox. This was also confirmed using highly sensitive anti-HA and GFP antibodies in Western blot analyses (Figure 2C**/D**). However, dox concentrations as little as 0.02 μg/ml induce transgene expression, while concentrations from 0.2 μg/ml induce an almost complete induction of transgene expression. We also noticed that a higher dox concentration did not further increase transgene expression levels, which is in agreement with dose-response curves established in HeLa cell lines stably transfected with rtTA2^S^-M2 (Ref. [16]). One potential advantage of conditional gene expression systems is that the level of the transgene can be titrated using various concentrations of the inducer compound. At a first glance, however, the data in Figure 2A suggest that our system behaves rather switch-like. Nevertheless, upon closer inspection, we noticed that cells responding to 0.02 μg/ml dox display a mean GFP fluorescence about half a log unit lower than those exposed to 0.2 μg/ml dox. These data indicate that, within certain limits, graded transgene expression can be achieved as it was also demonstrated with the pWHE644 plasmid in Jurkat T [15] and CaCo-2 cells (data not shown). Next, we evaluated the temporal kinetics of transgene expression. As shown in Figure 2B, GFP expression could be already detected 3 h following dox exposure and reached a plateau about 24 h later. Interestingly, the mean GFP fluorescence at 6 h was roughly ten times lower than that at 24 h, which means that different expression levels can be also achieved by distinct exposure times.

Another important advantage of reversible, conditional gene expression systems is that they can be used to study the decay of the protein-of-interest and the associated biological consequences. Thus, we asked whether transgene expression could be efficiently reversed by dox wash-out. Therefore, MCF-10A cultures were treated with dox for 24 h and then subjected to dox withdrawal. As indicated in Figure 2C, both transgene products of pTET/HAhBRAF-IRES-GFP-bsr disappear over the following days indicating that this system is very suitable to study protein decay rates. A Western blot analysis of the phosphorylation status of MEK, the direct substrate of B-Raf revealed that the induction of B-Raf or its oncogenic form resulted in a strong activation of this pathway (Figure 2D). Furthermore, its phosphorylation in the absence of dox or following wash-out was not elevated in cells harboring the pTET/HAhBRAF-IRES-GFP-bsr plasmids, which, given the potency of B-Raf^V600E^, further supports our notion that this expression system is tightly controlled. Most importantly, the activity of this pathway could be reversed by dox withdrawal within 6 days. Taken together, our data clearly show that MCF-10Atet cells represent a tightly controlled, but still highly efficient gene expression system that can be used to manipulate signaling pathways in a dynamic manner.

### Conditional B-Raf^V600E ^expression in 3D cultures of MCF-10A cells reveals the plasticity of the transformed phenotype

**Figure 3:**
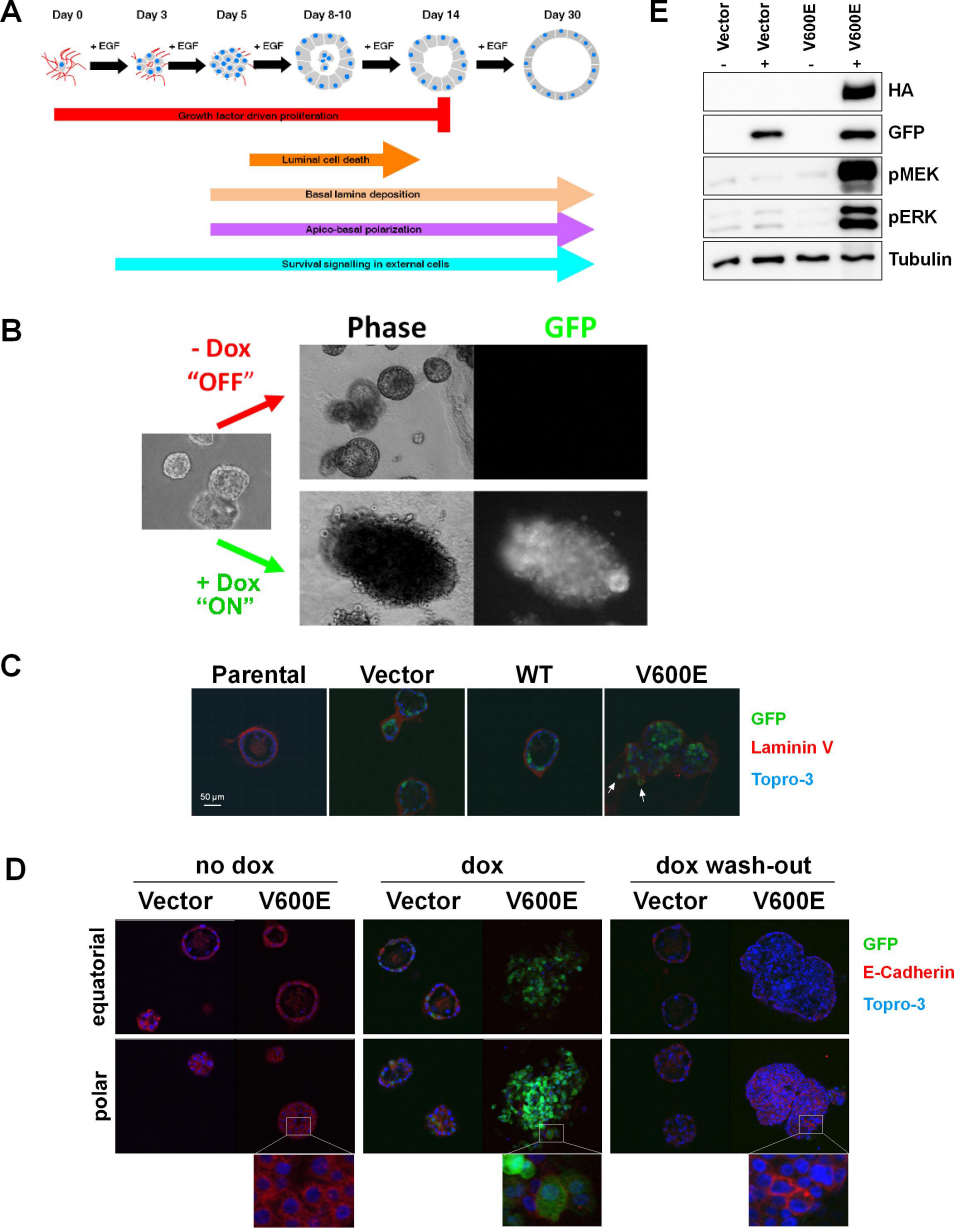
**Conditional Tet-regulated transgene expression in 3D cultures of MCF-10A cells**. **(A) **Overview on acinar development based on [1,7]. At day (d) 0, single MCF-10A cells are seeded into matrigel. Apico-basal polarization and luminal cell death occur after one week in culture. Two weeks later, hollow acini are surrounded by a thick, mostly single cell layer epithelium. During the third week, proliferation ceases, while the epithelium becomes thinner. **(B) **Expression of B-Raf^V600E ^in mid-term acini perturbs their morphogenesis. At d10, one culture was continued in the absence of dox, while the other was grown in the presence of dox for 14 days. **(C)**. The basal lamina of MCF-10A acini is disrupted by cells expressing oncogenic B-Raf^V600E^. Thirty-three days old acini grown in the presence of dox for 17 days were fixed and stained with an antibody for laminin V. Note the diffuse laminin V deposition surrounding cells expressing B-Raf^V600E ^(indicated by an arrow). **(D) **Dox withdrawal leads to a loss of invasive structures and reappearance of E-Cadherin. Three D cultures of MCF-10A cells harboring a dox-inducible B-Raf^V600E ^oncogene were seeded and switched to dox-containing medium at day 17 for 18 days. Then, dox was withdrawn from one culture (dox wash-out), while the other culture was kept under dox. Following another 24 days, the cultures were fixed and stained with anti-E-Cadherin antibodies (red) and Topro-3 (blue) to identify cell-cell contacts and nuclei, respectively. **(**E**)** Western blot analysis of 3D cultures showing tight regulation of the rtTA2^S^-M2/tTS^D^-PP expression system. MCF-10A cells harboring the inducible B-Raf^V600E ^or empty vector control constructs were induced at day 6 after seeding and harvested 4 days later.

Next, we sought to apply the inducible system to study how oncogenes affect the acinar morphogenesis of MCF-10A cells in 3D culture (Figure 3A). We have previously shown that oncogenic B-Raf^V600E ^induces the epithelial-to-mesenchymal transition (EMT) of MCF-10A cells and therefore, if expressed *a priori*, prevents acinar morphogenesis [25]. Consequently, we asked as to how the sudden expression of B-Raf^V600E ^would impinge on later stages of acinar morphogenesis and homeostasis. Therefore, MCF-10Atet cells stably transfected with pTET/HAhBRAF^V600E^-IRES-GFP-bsr plasmids were seeded into matrigel as described previously [8] and cultivated for 10 days (Figure 3B). In the absence of dox, cells harboring the indicated plasmids developed into regular acini characterized by radial symmetry and a thin, polarized and, based on previous work [7,14] and analyses shown below, most likely growth-arrested, epithelium consisting of a single cell layer. However, if the developing acini were exposed to dox from day 10 onwards, drastic morphological changes were observed over the next fortnight. The structures appeared very dense and optical sections using confocal laser scanning microscopy confirmed the absence of a lumen (Figure 3D). This indicates that acinar morphogenesis is disrupted by either unrestrained proliferation and/or reduced programmed cell death as it will be confirmed below. Importantly, some B-Raf^V600E ^expressing cells also protrude from the former acinus into the matrigel indicating that evading cells have destroyed the basal lamina (Figure 3B), which is being deposited from day 4 onwards [7]. Indeed, the typical laminin V coat, which marks the basal surface of MCF-10A acini and was supposedly deposited before dox exposure, is disrupted by GFP positive, B-Raf^V600E ^expressing cells (Figure 3C). This highly abnormal, invasive phenotype was present in more than 94% of GFP-positive acini (Additional data file 2). In full agreement with our earlier study showing that constitutive expression of B-Raf^V600E ^leads to a drastic reduction of E-Cadherin expression and the induction of other EMT hallmarks [25], we observed that inducing expression of this oncogene in 10 day old acini resulted in a loss of E-Cadherin expression in GFP-positive cells (Figure 3D). In contrast, acini harboring this oncogenic transgene, but not exposed to dox, develop into regular, symmetric, hollow and E-Cadherin positive structures, which are comparable to acini formed by doxycycline-treated cells transfected with the empty pTET/-IRES-GFP-bsr plasmid. This indicates that the corruption of acinar structure and development observed in the dox-treated, B-Raf^V600E ^expressing cells can be attributed to the effect of the oncoprotein and does not represent a side effect of doxycycline or GFP expression. Furthermore, it should be noted that acini, which harbor the *BRAF*^V600E ^transgene but were not exposed to dox, are in the same size range as the empty vector control acini and of comparable morphology. Given the profound effects of this oncogene on acinar development, this observation further indicates that our dual rtTA2^S^-M2/tTS^D^-PP expression system is tightly controlled. This notion is also strongly supported from our Western blot analysis showing the absence of HA-B-Raf and GFP in untreated 3D cultures (Figure 3E).

**Figure 4:**
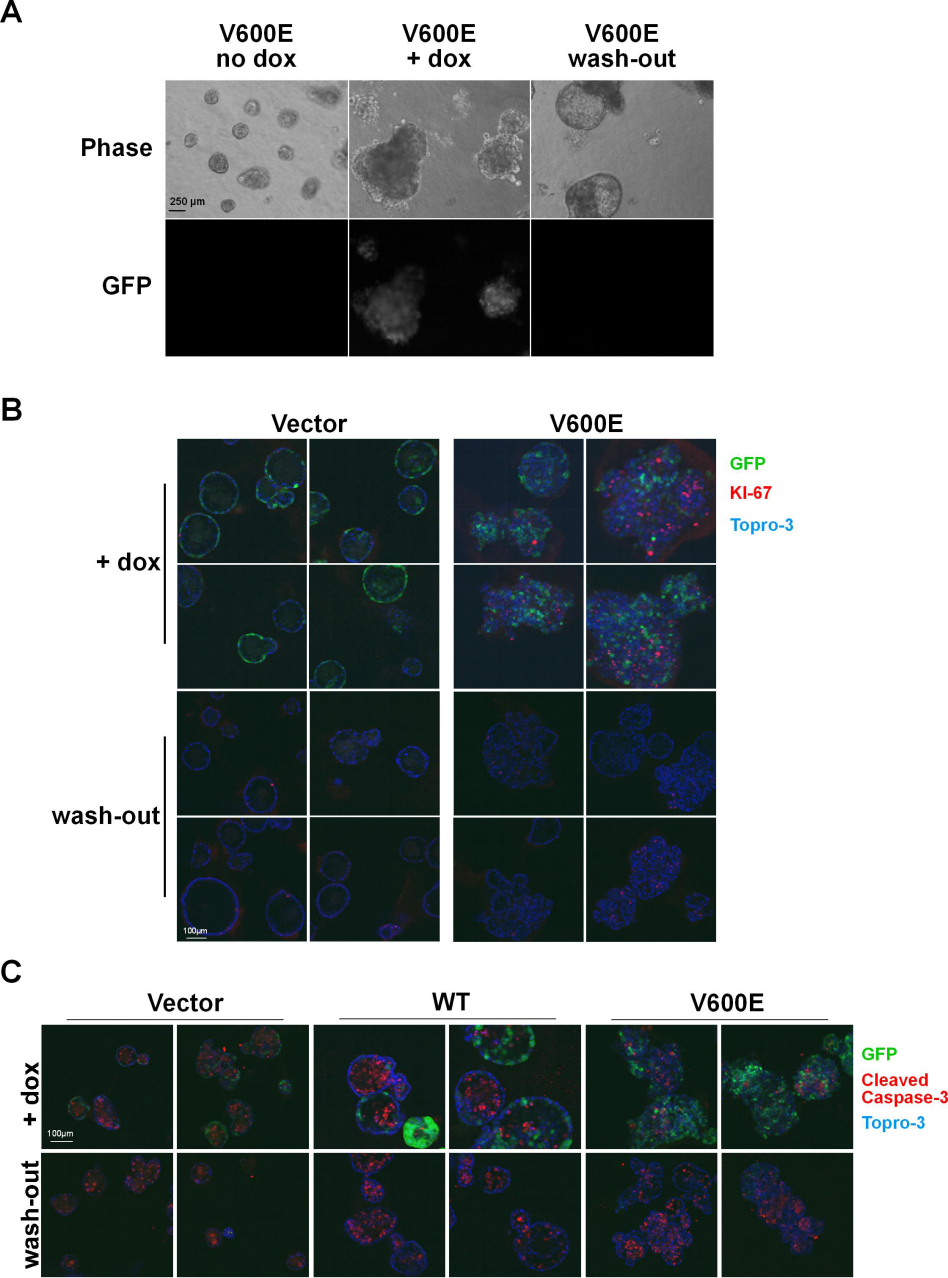
**Dox withdrawal restores acinar architecture of B-Raf^V600E ^transformed acini**. **(**A**)** Micrographs of MCF-10A acini harboring a B-Raf^V600E ^transgene in the absence (left), presence (middle) and following removal of dox (right panel). Representative phase contrast (upper row) and fluorescence (lower row) micrographs were taken at day 59. Each micrograph was taken using a 10x objective. Note the enlarged diameter of the acini in which B-Raf^V600E ^is or has been expressed. Note also the absence of invasive cells and the reappearance of a regular acinar architecture in acini following dox wash-out (right panel). **(**B**)** Representative confocal microscopy sections of 53 days old acini stained with the proliferation marker KI-67 (pink). **(**C**)** Representative confocal microscopy sections of 53 days old acini stained with the apoptotic marker cleaved Caspase-3 (pink). In both **B **and **C**, the nuclei were stained with Topro-3 (blue). Acini grown in the presence of dox appear green due to the expression of the GFP reporter.

Lastly, we were interested as to whether these profound changes in acinar homeostasis and development could be reversed by dox withdrawal. Consequently, MCF-10A cultures, which had been exposed to dox and displayed invasive behavior, were subsequently cultured in medium lacking dox. During the following days, we observed by visual inspection that the GFP expression disappeared, as one would anticipate based on our wash-out experiments in 2D culture (Figure 2C/D). Importantly, we noticed that signs of invasive cell behavior were less frequently observed over time and that acinar structures increasingly regained a smoother appearance and a more regular morphology. Twenty-four days following wash-out, the overwhelming majority of structures progressively resembled mature MCF-10A acini (Figure 4A), which eventually displayed prominent E-Cadherin deposition at baso-lateral cell-cell junctions again (Figure 3D). This indicates that the EMT inducing effect of B-Raf^V600E ^is reversible. However, we also observed that, as a result of the B-Raf^V600E ^driven increase in cellularity, these acinar structures were often enlarged, irregularly shaped and contained many nuclei in the re-developing lumen. Importantly, many of these nuclei displayed the typical morphology of fragmented nuclei derived from dead or dying cells, as it has been described for this model system previously [7]. In order to get more insight into the redevelopment process of acini following the withdrawal of B-Raf^V600E^, we stained 3D cultures grown in the absence or presence of dox as well as those subject to our induction/wash-out protocol with markers for proliferation (Figure 4B) and cell death (Figure 4C). Using KI-67, whose expression is strictly associated with cell proliferation [27], we show that late stage acini containing the "empty" pTET/-IRES-GFP-bsr control construct display hardly any KI-67 positive cells. This finding is in full agreement with previous studies on MCF-10A cells reporting the suppression of proliferation in mature acini [7,9,14,28,29] and further illustrates that MCF-10Atet cells and their sublines retain the typical characteristics of their MCF-10A and MCF-10AecoR predecessors. In sharp contrast, acini of MCF-10Atet/cells transfected with pTET/HAhBRAF^V600E^-IRES-GFP-bsr and exposed to dox display not only the abnormal morphology described above, but contain many KI-67 positive cells randomly scattered across the colony. Upon dox wash-out, however, KI-67 positive cells are hardly observed and only within those structures that still displayed higher degrees of irregularity (Figure 4B). This indicates that there are correlations between the maintenance of B-Raf^V600E ^expression, cell cycle progression and the restoration of acinar organization.

A previous study within the MCF-10A system has shown that luminal filling or the failure of luminal clearance is not only caused by stalled anti-proliferative programs, but also by the loss of luminal apoptosis [9]. Therefore, we also analyzed the extent of cell death using an antibody detecting the cleaved, activated form of caspase-3, a master regulator of apoptosis [7]. As shown in Figure 4C, MCF-10Atet cells transfected with the empty vector or with pTET/HAhBRAF^WT^-IRES-GFP-bsr display a large number of apoptotic cells in their presumptive lumina, regardless as to whether they have been exposed to dox or not at the time of fixation. In contrast, MCF-10Atet cells expressing B-Raf^V600E ^display some apoptotic cells scattered within the disintegrating acinus, but lack the display of a synchronized apoptosis in the lumen. These data are in agreement with the anti-apoptotic role of B-Raf^V600E ^[30] and suggest that luminal filling occurs in acini expressing this oncogene as a consequence of increased proliferation and impaired luminal apoptosis. However, as soon as B-Raf^V600E ^is withdrawn, the luminal apoptosis program, which depends on the establishment of apico-basal polarity and the coordination of intra- as well as supracellular signaling events [1,10,31-33], is restored (Figure 4C). Taken together, our findings demonstrate that the transformed 3D phenotype of B-Raf^V600E ^expressing acini can be efficiently reversed following the withdrawal of the oncogenic signal and that the morphogenetic program of acinus formation can be restored in late stage, highly disorganized MCF-10A acini.

## Discussion

Oncoproteins are dominant-positive acting versions of "cellular decision makers" that usually control cellular growth, survival, proliferation and differentiation. Consequently, the appearance of an oncoprotein often leads to a drastically distorted intracellular signaling network and an altered cellular phenotype. The conversion of a relatively immotile, epithelial cell into a highly motile mesenchymal cell with new features such as stem cell-like properties during EMT is a prime example for such a trans-differentiation process [3,34,35]. While EMT is regarded as a plastic and dynamic event during embryogenesis, the plasticity and reversibility of such cell fate alterations in tumorigenesis remains a matter of debate ([36,37] and references therein). So far, oncogenes have been mainly studied in the context of tumor cell initiation, but with the discovery of phenomena such as "oncogene addiction" and with the increasing portfolio of drugs targeting oncoproteins, a crucial question is how important the persistence of oncogene expression is for the maintenance of the transformed phenotype. In order to tackle this question, oncogene expression systems that can be regulated at will are of utmost importance for biomedical research.

By applying a novel and tightly controlled tetracycline-regulated gene expression system to MCF-10A cells, we believe that we have further improved this important model system, which is frequently used to study epithelial cell biology and early carcinogenic events alike. Firstly, this system allows the analysis of the presence/absence of the gene of interest in an isogenic setting. Thus, within the same genetic background, cells expressing high or low levels of the protein of interest can be compared with cells expressing no transgene product at all. Consequently, artefacts caused by clonal variability can be avoided. Furthermore, by establishing the MCF-10Atet line and our sequential transfection protocol (Figure 1C), we provide a model system, which can be used to generate several sublines in parallel that express various genes of interests independently. As such sublines are derived from the same parental cell line, this approach will allow for a better comparison of research results with previous experiments or with the work from other laboratories.

It should be noted that conditional systems have been implemented in MCF-10A cells before and have provided novel insights into the function of oncogenic receptor tyrosine kinases (RTKs). In this experimental setting, RTKs were fused with dimerization domains that can be cross-linked by an artificial ligand. However, this system relies strongly on the principle that RTKs often use dimerization as a mechanism for activation [28,38]. Similarly, a fusion protein consisting of a modified estrogen receptor (ER) binding domain and the isolated kinase domain of Raf-1 (ΔRaf-1:ER) has been used to activate the ERK pathway in the MEC lines MCF-10A and T4-2 [12,13] as well as in many other cell types [39,40]. As Raf-kinases are auto-inhibited by various structural features outside of their kinase domain [25,41], this fusion protein becomes constitutively and rapidly active upon 4-HT- or β-estradiol-mediated dissociation of the Hsp90 chaperone complex from the ER moiety and confers constitutive MEK/ERK signaling [39]. Thus, in the conditional systems employing ligand-induced RTK dimerization or exposure of a Raf-1 kinase domain, the proteins under scrutiny are expressed constitutively and must tolerate the fusion partner, e.g. a dimerization or ER domain. Furthermore, as in the case of 4-HT controlled Raf proteins [39,40], this strategy often requires the removal of large regulatory domains involved in auto-inhibition and other processes. Consequently, these modified proteins cannot exert the whole set of intra- and intermolecular interaction events like their full-length counterpart. With our inducible gene expression system, however, we can now provide a system that allows the expression of proteins lacking any modification, which is an important advantage for the conditional expression of cytoplasmic or nuclear proteins. While our manuscript was in preparation, Chen et al. reported the conditional expression of MLK3 in 3D cultures of MCF-10A cells using the ARGENT transcriptional regulation technology using the compound AP21967 (Ariad) as an inducer of gene expression [42]. As this system is independently regulated from the tetracycline-controlled expression system established in our study, one could envisage that the combinatorial use of both systems will allow the generation of MCF-10A cells harboring both systems. This would give cancer researchers additional flexibility to model multi-step oncogenesis and to delineate the cooperation of oncogenes.

Using B-Raf^V600E ^as a model oncogene, we have further substantiated that aberrant Raf-signaling counteracts lumen formation and confers invasive properties to MECs grown in 3D culture. We provide evidence that the impaired lumen formation in B-Raf^V600E ^expressing cells results most likely from the combination of a lack of proliferative suppression in late stage cultures and the failure to induce systematic luminal apoptosis. Our observations are in full agreement with previous studies showing that increased MEK/ERK signaling in 3D cultures of MCF-10A cells blocks luminal clearance by preventing the function and expression of the pro-apoptotic BIM protein, a critical regulator of acinar morphogenesis [43,44]. This notion is also in agreement with the observation that B-Raf^V600E ^is a potent suppressor of BIM in colorectal cancer models [30]. Likewise, the invasive properties are in line with our previous observation that constitutive expression of this oncoprotein induces EMT in MCF-10A cells [25] and with a report showing increased cellular motility in MCF-10A cells expressing ΔRaf-1:ER [12,45]. Furthermore, a recent report demonstrated the disruption of acinar architecture by ΔRaf-1:ER induced matrix metalloproteinase 9 (MMP9) expression [13]. Despite some phenotypic similarities, however, we feel that the invasive phenotype induced by B-Raf^V600E ^is more drastic than that induced by ΔRaf-1:ER. Firstly, this oncogenic Raf protein induces a complete EMT as judged by the loss of E-Cadherin and the gain of vimentin expression (Figure 2B and Ref. [25]). This was not observed in MCF-10A cells expressing ΔRaf-1:ER [12]. Secondly, the acinar structures, in which B-Raf^V600E ^has been induced, display an invasive behavior after a few days in that sense that individual cells protrude into the matrigel and thus most likely through the basement membrane (Figure 2C), which is deposited from day 4 onwards [1]. This observation, which was also not observed in MCF-10A acini expressing ΔRaf-1:ER [12,45], implies the induction of proteolytic processes by aberrant Raf signaling. This notion is also in line with the finding that MMP9 is a target of the Raf/MEK/ERK pathway [13] and given that B-Raf is a far more potent ERK activator than Raf-1 [39], it is conceivable that MCF-10A cells expressing oncogenic B-Raf produce higher levels of proteases compared to ΔRaf-1:ER expressing ones. Thus, it is not surprising that the disruption of acinar architecture, which is observed in MECs expressing ΔRaf-1:ER [12,13], is drastically exacerbated in MCF-10A cells expressing oncogenic B-Raf. These findings might also contribute to our understanding of the mechanisms by which aberrant Ras/Raf/MEK/ERK signaling is involved in the biology of basal type breast carcinomas [22,23].

## Conclusions

In conclusion, we have established an MCF-10A cell line fitted with a novel and tightly controlled tetracycline-regulated gene expression system. We believe this has further improved the application range of MCF-10A cells, an important model system to study epithelial cell biology and early carcinogenic events alike, for the following reasons: Firstly, this system allows the analysis of effects caused by the presence or absence of the gene-of-interest in an isogenic background and therefore avoids artifacts caused by clonal variability. Secondly, transgene expression can be efficiently reversed by dox withdrawal and consequently this system can be used to address the plasticity of the observed phenotype. Hence, these MCF-10Atet cells represent an important tool to understand phenomena such as EMT, oncogene addiction, oncogene-induced senescence and drug resistance.

## Methods

### Cell lines and tissue culture

Parental MCF-10A cells were obtained from the stock collection at the Garvan Institute of Medical Research, Sydney, and represent an early passage population from an import from ATCC. Its subline, MCF-10AecoR (a kind gift of Drs. Danielle Carroll and Joan Brugge; Harvard Medical School), has been described by various assays and in detail before [14,25,44,46-48]. MCF-10Atet cells were generated by transfecting MCF-10AecoR cells with the *Ahd*I-linearized expression vector pWHE644, which uses the EF-1α promoter [49] to express a tri-cistronic transcript encoding the tetracycline-regulated transcriptional activator rtTA2^S^-M2, the Tet-transsilencer tTS^D^-PP as well as a puromycin resistance gene [15]. Details of the construction of pWHE644 will be presented elsewhere (Danke et al., manuscript in revision). The cell lines in this study were generated by transfecting MCF-10AecoR or MCF-10Atet cells (2 × 10^7^/700 μl) in hypo-osmolar electroporation buffer (Eppendorf) with 30 μg *Ahd*I-linearized plasmid DNA by three electroporation pulses, each separated by a 1 min interval, using an Eppendorf multiporator set at 750 V and 70 μS. However, we have recently noticed that nucleofection of 2 μg *Ahd*I-linearized plasmid DNA and program T-024 using solution T (Amaxa) gave better transfection efficiencies than conventional electroporation (data not shown). Regardless of the transfection method, the cells were placed under selection using the appropriate antibiotics (puromycin (Sigma): 1.5 μg/ml; blasticidin S (Roth): 6 μg/ml) following a recovery period for 24 h in conventional growth medium. MCF-10A cells were set up for 3D culture experiments as described previously [14,25]. Fixation and staining of 3D cultures with the antibodies specified below was performed as described previously [14,25].

### Dox dose-response and time-course experiments

Dose-response experiments as shown in Figure 1D were carried out to identify the optimal dox concentration. MCF-10Atet cells stably transfected with either pTET-GFP-bsr (vector) or pTET-HA-B-Raf-GFP-bsr encoding HA-tagged B-Raf^wt ^were plated and allowed to adhere for 24 h. Subsequently, the cells were incubated with the indicated dox concentrations. After 24 h, they were harvested by trypsinization and subjected to FACS analysis using a CyAN cytometer (Beckman-Coulter) and standard gating procedures to exclude non-viable cells. Time-course experiments were set up as described for dose-response experiments using a dox concentration of 2 μg/ml. At the indicated time points, the cells were harvested and analyzed by FACS.

### Expression constructs

In order to generate an expression vector allowing the simultaneous, dox-inducible expression of hemagglutinin-tagged human B-Raf (HAhB-Raf) and green fluorescent protein (GFP), the HAh*BRAF*-IRES-GFP cassettes were amplified by PCR from the retroviral pMIG/HAh*BRAF*^wt ^or pMIG/HAh*BRAF*^V600E ^expression vectors [50] using oligonucleotides matching regions 5' and 3' of this cassette and the proof-reading polymerase Phusion (Finnzymes). These oligonucleotides introduce each a *Not*I site into the PCR amplicon. The PCR amplicons were then subcloned into the cloning vectors pBSSK+ or pSCA-Amp/Kan (Stratagene) for further propagation. The subcloned amplicon was then recovered by *Not*I digestion and subcloned into *Not*I-linearized pTET-bsr. This plasmid is derived from pWHE636 and contains as novel features a *Not*I site in its multicloning site and a *lox*P-flanked blasticidin S resistance (bsr) cassette derived from pA*lox*P-bsr [51]. pWHE636 is based on the Tet-response vector pUHD10-3 (Ref. [52]) and contains, instead of the original promoter *P*_TRE_, the second generation promoter *P*_SG-TRE _(Ref. [53]) to drive expression of a cDNA encoding EGFPmut2 (Ref. [54]). Detailed cloning procedures, as well as plasmid sequences, are available upon request. In brief, pWHE636 was digested with *Nco*I and *BamH*I to remove the existing EGFPmut2 cassette and the staggered cuts in the vector backbone were blunted by *Pfu *polymerase treatment. Subsequently, the vector was re-ligated and a *Not*I site was introduced downstream of the CMV minimal promoter by conventional site-directed mutagenesis PCR. Subsequently, a *lox*P-flanked blasticidin S resistance gene cassette under the control of the chicken β-actin promoter was excised from pAloxP-bsr [51] by *BamH*I digestion, blunted by *Pfu*-polymerase treatment and subcloned into the *Mfe*I-linearized, blunted pWHE636 backbone to yield pTET-bsr.

### Antibodies, biochemical analysis and Western blotting

Two D and 3D cultures of MCF-10A cells were processed to total cellular lysates and analyzed by Western blotting as described previously [25]. Lysates from 3D cultures were prepared using the Becton Dickinson (BD) cell recovery solution. Antibodies used in this study were anti-E-Cadherin (clone 36; BD Transduction Laboratories, Heidelberg, Germany), anti-phospho-MEK (pMEK), anti-MEK1/2, anti-phospho-p42/44 (pERK), anti-Akt, anti-cleaved Caspase-3 (Asp175) (from Cell Signaling Technologies, Frankfurt, Germany) anti-β-Actin (C4), anti-α-Tubulin (from Santa Cruz Biotechnologies, Santa Cruz, CA, USA), anti-HA antibody 3F10, anti-GFP (from Roche Molecular Bioscience, Mannheim, Germany), anti-human Epiligrin (Laminin V) (Millipore, Schwalbach, Germany), anti-KI-67 (clone MIB-1; from Dako) and the secondary antibodies Alexa Fluor^® ^546 goat anti-rabbit IgG, Alexa Fluor^® ^488 goat-anti rabbit IgG and Cy3 goat anti-mouse IgG (from Invitrogen, Darmstadt, Germany). The polyclonal antibody against the Tet-transregulators was generated in-house in the laboratory of Dr. Christian Berens.

### Flow cytometry

FACS-analysis was performed to measure GFP expression in cells transfected with IRES-based bicistronic vectors. Cells were harvested by standard trypsinization [8] as described previously. The pellet was resuspended in 300 μl FACS buffer (phosphate-buffered saline supplemented with 3% fetal calf serum and 0.1% sodium azide). GFP fluorescence was examined by flow cytometry using a CyAN ADP flow cytometer (Beckman-Coulter). The 488 nm laser was used for excitation and fluorescence was detected in channel FL-1, respectively.

## Abbreviations

**3D**: Three dimensional; **4-HT**: 4-hydroxy-tamoxifen; **BSR**: Blasticidin S resistance, **CMV**: Cytomegalovirus; **DCIS**: Ductal carcinoma in situ; **Dox**: Doxycycline; **EGFP**: Enhanced green fluorescent protein; **EMT**: Epithelial-to-mesenchymal transition; **ER**: Estrogen receptor; **ERK**: Extracellular signal-regulated kinase; **FACS**: Fluorescence activated cell sorter; **GFP**: Green fluorescent protein; **HA**: Hemagglutinin; **IRES**: Internal ribosomal entry site, **MECs**: Mammary epithelial cells; **MMP**: Matrix metalloproteinase; **Raf**: rapidly growing fibrosarcoma; **Ras**: Rat sarcoma; **rtTA**: Reverse tetracycline-dependent trans-activator; **PCR**: Polymerase chain reaction; **RTK**: Receptor tyrosine kinase; **Tet**: Tetracycline; **tTS**: Tetracycline-dependent trans-silencer; **MEK**: Mitogen-activated protein/extracellular signal-regulated kinase (ERK) kinase.

## Competing interests

The authors declare that they have no competing interests.

## Authors' contributions

RH performed all the functional assays in the manuscript and generated the MCF-10Atet cell line. RH, FUW, CD, CB and TB generated the novel expression constructs and/or were involved in their design. RH und FUW performed confocal microscopy. All authors contributed to data analysis. The manuscript was written by TB with input from RH, FUW and CB. All authors read and approved the manuscript.

## Supplementary Material

Additional file 1 **Regulatory properties of the cell lines Jr2^S^M2K and Jr2^S^M2PP**. Jurkat cells were stably transfected with pWHE459 or pWHE644 resulting in the regulator lines Jr2^S^M2K and Jr2^S^M2PP, both expressing the transactivator variant rtTA2S-M2 and either the transrepressor tTS^D^-KRAB or tTS^D^-PP, respectively. To test their respective regulatory capacity, single-cell clones of each cell line were transiently transfected with 800 ng pUHC13-3, coding for firefly luciferase under Tet-control. For each cell line, three well-regulating clones are depicted above. The basal luciferase activity obtained in likewise transfected Jurkat cells not containing any transregulators is shown on the right and serves as control for repression by tTS and activation of transgene expression by rtTA. Click here for file

Additional file 2 **Quantification of normal and aberrant MCF-10A acini**. Cells harboring dox-inducible B-Raf^WT^, B-Raf^V600E ^and vector control constructs were seeded into matrigel, induced at day 17 and subjected to dox withdrawal at day 29. Representative micrographs were taken at day 59. At least 34 acini were counted and categorized into non-invasive phenotype and invasive phenotype (minimum three cells protruding from the acinus). Click here for file
