# Supplementary figures and images for: A novel MCF-10A line allowing conditional oncogene expression in 3D culture
Source: Cell Commun Signal. 2011 Jul 13;9:17. doi: 10.1186/1478-811X-9-17 (PMC3163222; doi:10.1186/1478-811X-9-17)

## Slide 1
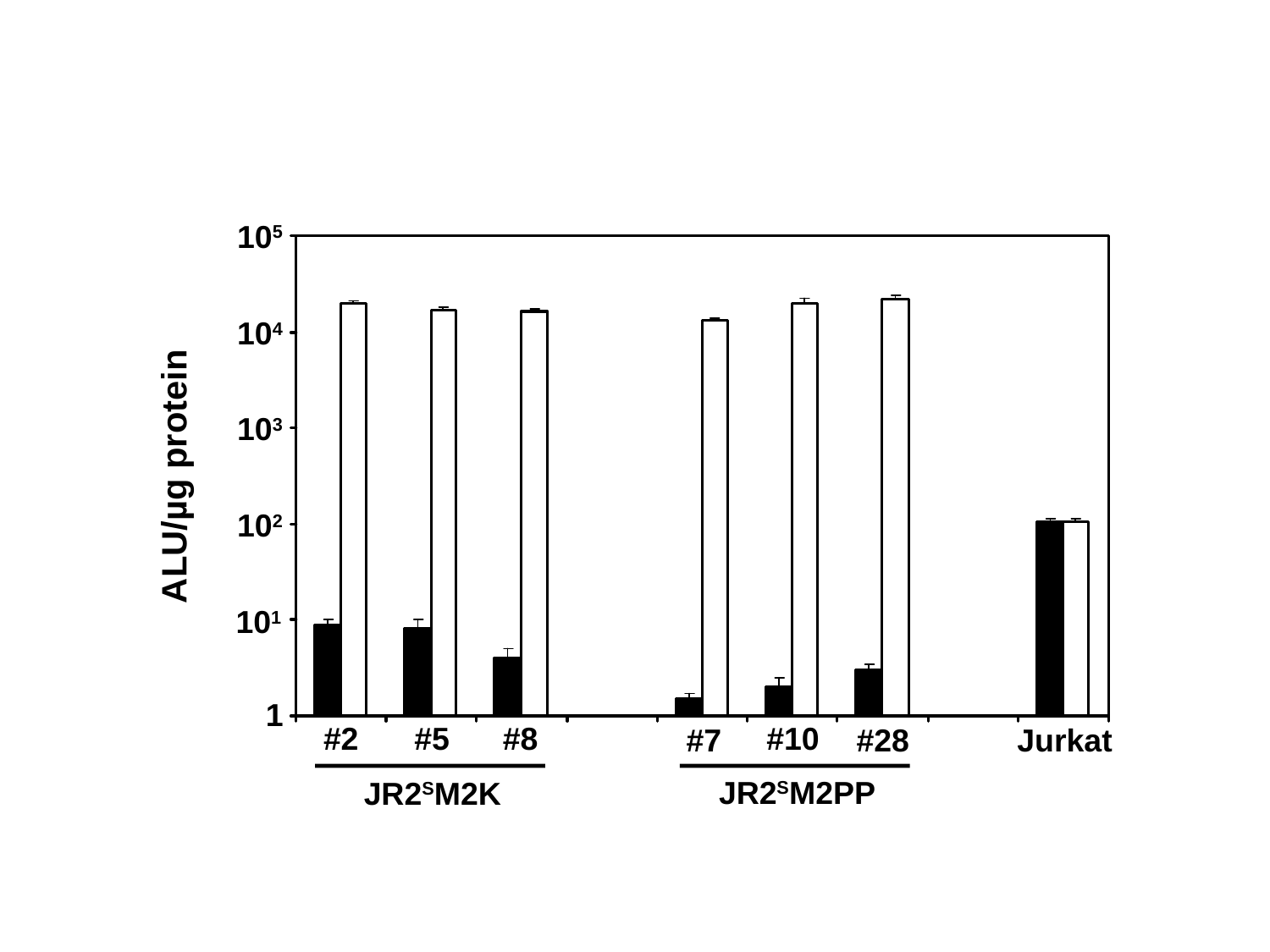

105
104
103
ALU/µg protein
102
101
1
#5
#2
#8
#10
#7
#28
Jurkat
JR2SM2PP
JR2SM2K

Supplement: Additional file 1 — Regulatory properties of the cell lines Jr2SM2K and Jr2SM2PP. Jurkat cells were stably transfected with pWHE459 or pWHE644 resulting in the regulator lines Jr2SM2K and Jr2SM2PP, both expressing the transactivator variant rtTA2S-M2 and either the transrepressor tTSD-KRAB or tTSD-PP, respectively. To test their respective regulatory capacity, single-cell clones of each cell line were transiently transfected with 800 ng pUHC13-3, coding for firefly luciferase under Tet-control. For each cell line, three well-regulating clones are depicted above. The basal luciferase activity obtained in likewise transfected Jurkat cells not containing any transregulators is shown on the right and serves as control for repression by tTS and activation of transgene expression by rtTA. [file 1478-811X-9-17-S1.PPT]

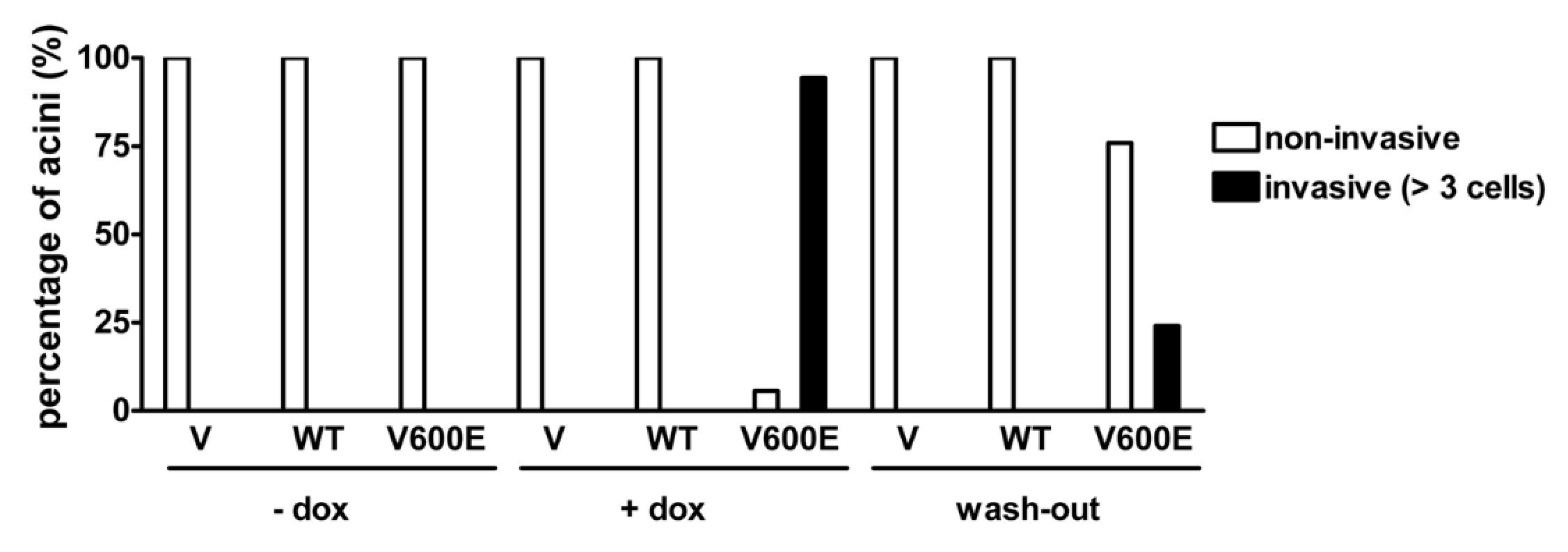

Supplement: Additional file 2 — Quantification of normal and aberrant MCF-10A acini. Cells harboring dox-inducible B-RafWT, B-RafV600E and vector control constructs were seeded into matrigel, induced at day 17 and subjected to dox withdrawal at day 29. Representative micrographs were taken at day 59. At least 34 acini were counted and categorized into non-invasive phenotype and invasive phenotype (minimum three cells protruding from the acinus). [file 1478-811X-9-17-S2.JPEG]
